# Gaze Behavior of Children with ASD toward Pictures of Facial Expressions

**DOI:** 10.1155/2015/617190

**Published:** 2015-05-19

**Authors:** Soichiro Matsuda, Yasuyo Minagawa, Junichi Yamamoto

**Affiliations:** ^1^Department of Psychology, Keio University, 2-15-45 Mita, Minato-ku, Tokyo 108-0073, Japan; ^2^Japan Society for the Promotion of Science, 5-3-1 Kojimachi, Chiyoda-ku, Tokyo 102-0083, Japan; ^3^CREST, Japan Science and Technology Agency, 4-1-8 Honcho, Kawaguchi-shi, Saitama 332-0012, Japan

## Abstract

Atypical gaze behavior in response to a face has been well documented in individuals with autism spectrum disorders (ASDs). Children with ASD appear to differ from typically developing (TD) children in gaze behavior for spoken and dynamic face stimuli but not for nonspeaking, static face stimuli. Furthermore, children with ASD and TD children show a difference in their gaze behavior for certain expressions. However, few studies have examined the relationship between autism severity and gaze behavior toward certain facial expressions. The present study replicated and extended previous studies by examining gaze behavior towards pictures of facial expressions. We presented ASD and TD children with pictures of surprised, happy, neutral, angry, and sad facial expressions. Autism severity was assessed using the Childhood Autism Rating Scale (CARS). The results showed that there was no group difference in gaze behavior when looking at pictures of facial expressions. Conversely, the children with ASD who had more severe autistic symptomatology had a tendency to gaze at angry facial expressions for a shorter duration in comparison to other facial expressions. These findings suggest that autism severity should be considered when examining atypical responses to certain facial expressions.

## 1. Introduction

Human faces provide important social cues, and the perception of facial expressions is fundamental to the development of social communication. Therefore, it is expected that individuals with autism spectrum disorders (ASDs), who are characterized by social communication difficulties, exhibit abnormalities in their perception of the face. Consistent with this view, several eye-tracking studies have shown atypical gaze behavior toward faces in individuals with ASD [1–3]. Certain other studies, however, have not found these abnormalities [4, 5]. Matsuda and Yamamoto [6–8] suggested that these mixed results were caused by the differing stimuli, ages, and/or autism severity of the participants.

When considering the face as a stimulus, we need to recognize that there are several variables which might affect gaze behavior [9, 10]. Gaze behavior toward the face differs because of variables such as speech (spoken or unspoken), movement (static or dynamic), or facial expression. However, in most previous investigations of gaze behavior toward faces in individuals with ASD, these variables have not been sufficiently examined.

It is possible that atypical gaze behavior toward a nonspeaking static facial expression in individuals with ASD could be related to age. Adults with ASD have exhibited shorter fixation durations on the eyes or longer fixation durations on the mouth than those with typical development [11–17]. However, studies of children or adolescents with ASD have not found such differences [18–20] and have indicated that children with ASD have the same gaze behavior as typically developing children for facial expressions.

In typical development, there is evidence that gaze behavior varies in response to different expressions. Infants and adults were found to look for a shorter duration at a feature area with fearful and angry facial expressions in comparison to neutral and sad expressions [21]. Adults looked longer at the mouth for happy expressions compared to sad, neutral, and fearful facial expressions [22] and longer at the feature area for fearful and angry expressions compared to happy, sad, and neutral expressions [23].

Recent eye-tracking studies in individuals with ASD have demonstrated that differences in gaze behavior in certain expressions were also observed in this population. van der Geest et al. [19] demonstrated that children with ASD looked longer at the mouth for surprised and happy expressions compared to angry and neutral expressions. Wagner et al. [20] also found that adolescents with ASD looked longer at the mouth for happy expressions compared to neutral and fearful facial expressions. In both studies, there was no significant difference between individuals with and without ASD for each of the facial expressions.

However, although previous research has suggested that autism severity is linked to fixation duration on the eyes or the mouth [2, 18, 24], no study has examined the relationship between autism severity and gaze behavior toward certain facial expressions. Some studies have reported that autism severity is negatively correlated with the percentage of correct responses in the naming of sad facial expressions [25], or fear facial expressions [26], which suggests that certain facial expressions may evoke a differential gaze behavior depending on autism severity.

In the current study, we presented children with ASD and typically developing children with pictures of surprised, happy, neutral, angry, and sad facial expressions. The participants' gaze behavior towards the facial expressions was assessed using an eye-tracking device. To investigate the role of autism severity in gaze behavior towards facial expressions, we examined the relationship degree between the total CARS scores (Childhood Autism Rating Scale) [27] and the proportion of total time spent looking at each facial expression.

## 2. Method

### 2.1. Participants

The participants included two groups of children: 18 with ASD and 11 typically developing (TD) children. Children in the ASD group had previously received a formal ASD diagnosis by an outside professional (one with Asperger's syndrome, 15 with autism, and two with Pervasive Developmental Disorders), according to the DSM-IV-TR criteria [28]. Children in the TD group were matched by their average age to the developmental age (DA) of those in the ASD group. Data analyses were conducted on a final sample of 15 boys with ASD (CA: *M* = 7.5, SD = 3.0, range: 3.6–13.7; DA: *M* = 5.1, SD = 2.0, range: 2.2–8.7) and 11 TD (CA: *M* = 5.5, SD = 1.8, range: 3.2–9.9; 3 female, 8 male) children. Three children with ASD were excluded from the analyses because they did not meet the CARS criteria for autism.

Developmental ages were derived using the Kyoto Scale of Psychological Development 2001 (KSPD) [29]. This assessment calculates the average scores of subitems for physical-motion, verbal-social, cognitive-adaptation, and total developmental age. The KSPD has been developed for typically developing infants and low-functioning children with ASD and other developmental disorders. In this study, the measure for the developmental age of the children was the total developmental age score in the KSPD.

Gold standard diagnostic measures, such as the Autism Diagnostic Interview−Revised [30, 31] and the Autism Diagnostic Observation Schedule [32], have not been officially translated and adopted to Japanese culture. Therefore, to confirm the diagnosis of ASD, we used the Japanese version of Childhood Autism Rating Scale [27]. A licensed psychologist and four therapists, who had at least five years' experience in the fields of behavior interventions, administered the CARS before the experiment. The mean autism severity of the children with ASD was 40.9 (SD = 6.5, range: 30.5–52.5). Autism severity was not associated with the CA (*r* = 0.55, *p* = 0.035, n.s. with Bonferroni correction) or DA (*r* = −0.34, *p* = 0.217).

The difference between the mean chronological age (CA) of each group approached significance; the participants in the ASD group were older than the participants in the TD group (*t*(24) = 2.03, *p* = 0.054). There were no significant differences between the mean DA in the ASD group and the CA in the TD group (*t*(24) = −0.40, *p* = 0.693).

### 2.2. Stimuli and Apparatus

The stimuli were color images of three female and two male Japanese actors' faces, each depicting a surprised, happy, neutral, angry, or sad expression. There were 25 images in total, with each image showing a single actor with one expression. All images were transformed with the Adobe Photoshop CS5 (Adobe, San Jose, CA) to control luminance, background colors, and face size. The emotions displayed in the images were selected from the list of six universal emotions identified by Ekman [33]: surprise, happiness, anger, sadness, fear, and disgust. In addition, a neutral expression was added. Emotions of fear and disgust were excluded from this study because Japanese participants in previous studies [34, 35] had had difficulty in identifying them. Prior to the study, a pilot study was conducted, in which 11 Japanese adults rated pictures of facial expressions. They were asked to rate the expressions on a 7-point Likert scale for each given emotion and the Affect Grid [36] for pleasure and arousal. The results of these ratings are presented in Table 1.

Participants sat in a chair facing the monitor in a testing room. With an approximately 75-cm viewing distance, the images (1024 × 731 px) measured approximately 24.0° and 17.3° of the vertical and horizontal visual angles, respectively. Eye-tracker calibration and stimulus presentation were controlled by Tobii Studio software (Tobii Technology).

Participant fixations were recorded using a Tobii X120 (Tobii Technology Japan, Ltd, Minato-ku Takanawa, Japan) at 60 Hz. Tobii has infrared light sources and cameras and uses corneal reflection techniques. Fixations were defined by an in-built automatic fixation detection algorithm that uses a sliding window average method (Tobii Fixation Filter). The velocity threshold was 35 pixel/window, and distance threshold was 35 pixels. The accuracy of this eye-tracker is approximately 0.5°. The eye-tracker was placed in front of a 27-inch monitor (1080 × 1920 px), which measured approximately 25.3° and 43.5° of the vertical and horizontal visual angles, respectively.

### 2.3. Procedure

The experiment was conducted in a testing room at Keio University. The children were asked to sit in a chair in front of the monitor and instructed that they will be shown some pictures and movies on the monitor. Prior to testing, the gaze of each child was calibrated. We used a 5-point calibration procedure, in which a movie clip (i.e., a moving cat coupled with attractive sounds) was played sequentially at five locations on the screen. If all five points were calibrated successfully, the experimental phase began. The accuracy of the calibration was similar in the ASD and TD group. In this phase, all 25 images were shown for three seconds each, with an attention getter appearing before every five images. There was no interstimulus interval between five images. The attention getter was a 3-second movie clip (i.e., a moving star coupled with attractive sounds). The image order was pseudo-randomized.

### 2.4. Data Analysis

#### 2.4.1. Areas of Interest (AOI)

**Figure 1:**
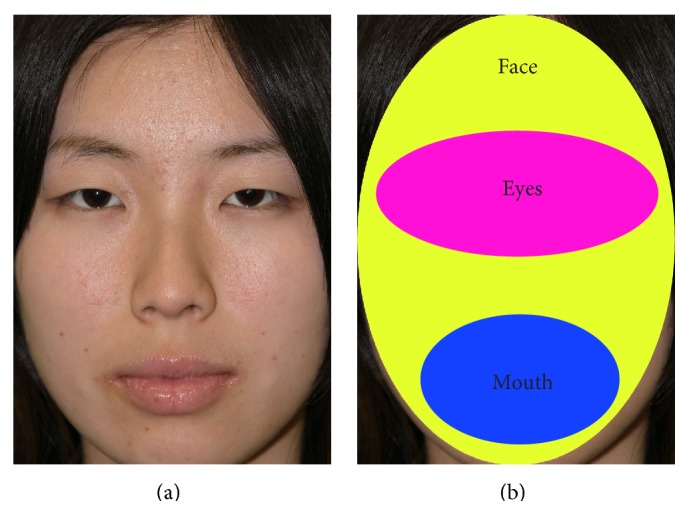
Visual example of the (a) stimuli and (b) areas of interest (AOI).* Note*. This is printed with permission from the model.

Three areas of interest (AOI) were manually defined: the face, eyes, and mouth. The face AOI covered the AOIs of both the eyes and the mouth, with the AOIs being equal for all images. The size of the AOIs was 78.1% of the image of the face AOI (24.0° and 17.2° of the vertical and horizontal visual angles), 19.1% for the image of the eyes AOI (15.5° and 6.1° of the vertical and horizontal visual angles), and 14.1% for the image of the mouth AOI (10.3° and 6.0° of the vertical and horizontal visual angles). An example of an image and AOIs are shown in Figure 1.

#### 2.4.2. Statistical Analysis

All analyses were conducted using SPSS for windows, version 22. The first set of analyses examined the degree to which the children with ASD displayed different gaze behavior toward the facial expressions compared to the typically developing children. Repeated measures ANOVAs with the facial expression as the within-subject factor (surprised, happy, neutral, angry, and sad) and diagnosis as the between-subject factor (ASD, TD) were conducted for the dependent variables. We also examined the significant effects and interactions using post hoc *t*-tests with a Bonferroni correction. The dependent variables were based on the proportion of the fixation duration. The proportion of the total time spent looking at the face AOI (%Face) was standardized using the total duration of the stimulus display, and the proportion of the total time spent looking at the eyes AOI (%Eyes) and the mouth AOI (%Mouth) was standardized using the total time spent looking at the face AOI.

The second set of analyses explored the relationship degree between CA, DA, or autism severity (the total CARS scores) and the gaze behavior toward each expression using Pearson correlations. The gaze behavior toward each facial expression was calculated for each child by dividing the fixation duration on the face AOI for each expression (e.g., surprised) by the total fixation duration on the face AOI for all expressions (surprised, happy, neutral, angry, and sad).

## 3. Results

### 3.1. Total Time Spent Looking at the Face

The mean proportion of the total time spent looking at the face AOI was 65.1% (SD = 18.7) for children with ASD and 77.5% (SD = 20.0) for TDs. The diagnosis × facial expression ANOVA on % Face revealed no effects for diagnosis, *F*(1, 24) = 2.49, *p* = 0.128, *ηp* ^2^ = 0.09, facial expression, *F*(4, 96) = 1.05, *p* = 0.384, *ηp* ^2^ = 0.04, or diagnosis × facial expression interaction, *F*(4, 96) = 0.31, *p* = 0.869, *ηp* ^2^ = 0.01.

### 3.2. Eyes and Mouth Ratios

**Figure 2:**
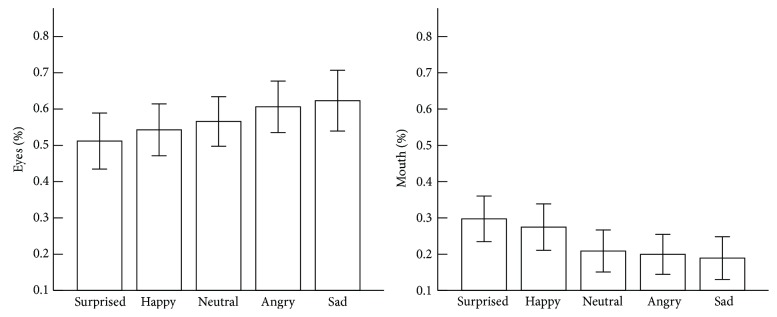
Mean proportion of total time spent looking at the eyes AOI and the mouth AOI for each facial expression category. The error bars indicate 95% confidence intervals.

Figure 2 shows the mean % Eyes and % Mouth for each facial expression category.

The diagnosis × facial expression ANOVA on % Eyes indicated a significant effect for facial expression, *F*(4, 96) = 5.01, *p* = 0.001, *ηp* ^2^ = 0.17, but no effect for diagnosis, *F*(1, 24) = 0.80, *p* = 0.380, *ηp* ^2^ = 0.03, or diagnosis × facial expression interaction, *F*(4, 96) = 0.27, *p* = 0.895, *ηp* ^2^ = 0.01. Post hoc *t*-tests for the main facial expression effects revealed significantly shorter fixation durations on the eyes for the surprised expression compared to the sad expression, *t*(25) = −3.61, *p* = 0.001, and the angry expression, *t*(25) = −3.31, *p* = 0.003. Both groups tended to look for a shorter time at the eyes for the happy expression compared to the sad expression, *t*(25) = −2.83, *p* = 0.009 (n.s. with Bonferroni correction).

The diagnosis × facial expression ANOVA for % Mouth indicated a significant effect for facial expression, *F*(4, 96) = 8.54, *p* < 0.001, *ηp* ^2^ = 0.26, but no effect for diagnosis, *F*(1, 24) = 0.57, *p* = 0.457, *ηp* ^2^ = 0.02, or diagnosis × facial expression interaction, *F*(4, 96) = 0.38, *p* = 0.823, *ηp* ^2^ = 0.02. Post hoc *t*-tests for the main facial expression effects revealed significantly longer fixation durations on the mouth for the surprised expression compared to the sad, *t*(25) = 4.47, *p* < 0.001, angry, *t*(25) = 4.57, *p* < 0.001, and the neutral, *t*(25) = 3.27, *p* = 0.003, expressions. In addition, both groups looked longer at the mouth for the happy expression compared to the sad, *t*(25) = 3.57, *p* = 0.001, angry, *t*(25) = 3.05, *p* = 0.005, and neutral, *t*(25) = 2.60, *p* = 0.015 (n.s. with Bonferroni correction), expressions.

### 3.3. Associations between CA, DA, and Fixation Durations for Each Facial Expression in ASD

CA was not associated with the proportion of total time spent looking at the surprised (*r* = 0.29, *p* = 0.104); happy (*r* = −0.10, *p* = 0.735); neutral (*r* = 0.02, *p* = 0.959); angry (*r* = −0.38, *p* = 0.167); or sad (*r* = −0.15, *p* = 0.603) expressions. DA was also not associated with the proportion of total time spent looking at the surprised (*r* = −0.10, *p* = 0.735); happy (*r* = 0.12, *p* = 0.957); neutral (*r* = 0.10, *p* = 0.717); angry (*r* = 0.20, *p* = 0.466); or sad (*r* = −0.18, *p* = 0.518) expressions.

### 3.4. Associations between Autism Severity and Fixation Durations for Each Facial Expression in ASD

**Figure 3:**
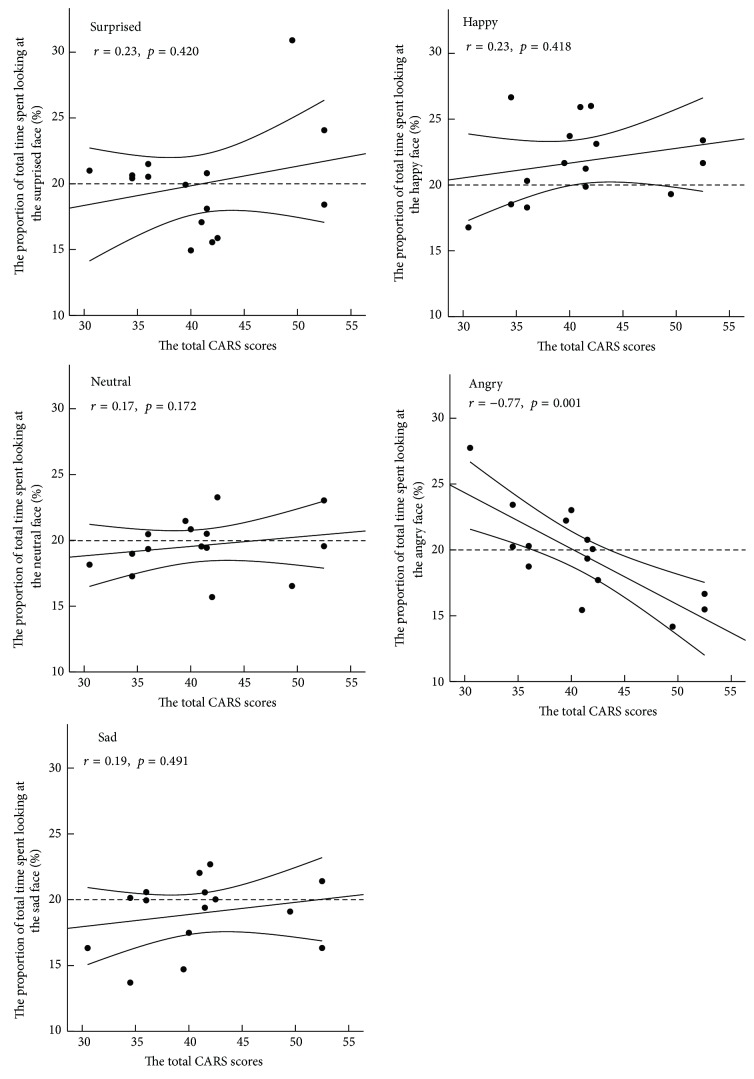
Correlation of the total CARS scores and the proportion of total time spent looking at each facial expression in ASD group.

Finally, we examined the correlation between autism severity and the proportion of total time spent looking at each facial expression. Autism severity was not associated with the proportion of total time spent looking at the surprised (*r* = 0.23, *p* = 0.420), happy (*r* = 0.23, *p* = 0.418), neutral (*r* = 0.17, *p* = 0.172), or sad (*r* = 0.19, *p* = 0.491) expressions. On the other hand, there was a significant negative correlation between autism severity and the proportion of total time spent looking at the angry expression, *r* = −0.77, *p* = 0.001 (Figure 3).

## 4. Discussion

In the current study, we examined gaze behavior in children with ASD and TD toward pictures of facial expressions. Overall, we found no group differences in gaze behavior toward the pictures of facial expressions. Further, the results of total time spent looking at the face and the proportion of time spent looking at the eyes and the mouth regions revealed no differences between children with ASD and TD. In both groups, the eyes of the angry and sad facial expressions were fixated on longer than the eyes of the surprised, and the mouth of the surprised and happy facial expressions was fixated on longer than the mouth of the angry and sad facial expressions. CA and DA were not associated with the proportion of total time spent looking at all facial expressions. While autism severity was not found to be associated with the proportion of total time spent looking at the surprised, happy, neutral, or sad facial expressions, it was found to be associated with a lower proportion of total time spent looking at the angry facial expression.

Our study demonstrated that there was no difference in gaze behavior between children with ASD and TD toward nonspeaking static facial expressions. This result is in accordance with recent eye-tracking studies of children and adolescents with ASD [18–20]. Although other eye-tracking studies on children or adolescents with ASD have found differences between ASD and TD [1, 3], one implication of this finding is that the presence of speech (spoken) and movement (dynamic) might be responsible for such differences.

It has regularly been reported that typically developing children show a specific fixation pattern when looking at certain facial expressions [21–23]. Several studies using visual search tasks have also demonstrated that mouth region had importance for surprised and happy expression, while eye region played important role for angry and sad expressions [37, 38]. The present study indicates that children with ASD have the same gaze behavior toward each facial expression. This result is in line with previous studies, which suggests that certain facial expressions do not cause atypical gaze behavior in children with ASD [19, 20].

Even though there were no group differences in gaze behavior toward certain facial expressions, the proportion of total time spent looking at the angry facial expression was found to be associated with autism severity. This result suggests that children with ASD who have a more severe autistic symptomatology tend to look for a shorter duration on angry facial expressions in comparison to other facial expressions. Children with ASD show atypical responses to angry faces on a visual search task [39], although adults with ASD show a similar response to those with a typical development [40, 41]. It is possible that both the autism severity and the participant ages may affect the atypical response to an angry facial expression.

There were several limitations to the study. First, we need to be cautious about interpreting lack of significant results between groups due to small sample size. Studies including more participants are needed. Second, the two groups differed slightly in CA. Future studies should include a CA matched control group to confirm that CA is not an important factor for lack of significant difference between two groups. Third, we used CARS to determine both eligibility and severity in this study. Further studies will be required to use other diagnostic measures in order that eligibility and severity are separately determined. Also, the ASD and TD groups were not matched on participant's gender.

Despite limitations, to our knowledge, this study is the first to demonstrate relationships between autism severity and gaze behaviors toward facial expressions. While previous studies examine the association between autism severity and fixation duration on the eyes or the mouth [2, 18, 24], our findings showed that angry expressions evoke a differential gaze behavior depending on autism severity. The current study contributes to a growing body of evidence that the demographic factor, including autism severity, affects the results of eye-tracking studies.

## 5. Conclusion

The present data suggested that there is no difference between children with ASD and TD when looking at a picture of a facial expression. Both groups showed differences in gaze behavior for certain expressions. That is, both groups looked longer at the eyes for the angry and sad facial expressions compared to the happy and surprised facial expressions. Furthermore, the results suggested that an atypical response to an angry facial expression might be because of autism severity.

## Figures and Tables

**Table 1:** Mean ratings for emotions, pleasure, and arousal for a stimuli based on a 7-point scale and an affect grid (9-point scale).

	7-point Likert scale				The Affect Grid (9-point)	
	Surprised	Happy	Angry	Sad	Pleasure	Arousal
Surprised	*5.6 *	1.6	1.5	1.5	3.9	6.7
Happy	1.5	*5.3 *	1.2	1.3	6.4	4.8
Neutral	1.5	1.7	2.0	2.3	3.6	3.6
Angry	1.6	1.2	*5.0 *	3.1	1.3	5.4
Sad	1.7	1.2	2.2	*4.3 *	1.9	3.3

*Note.* Ratings for predicted emotion are shown in italics.
